# Comprehensive Prevention and Control of Mastitis in Dairy Cows: From Etiology to Prevention

**DOI:** 10.3390/vetsci12090800

**Published:** 2025-08-23

**Authors:** Wenjing Yu, Zixuan Zhang, Zhonghua Wang, Xueyan Lin, Xusheng Dong, Qiuling Hou

**Affiliations:** College of Veterinary Medicine, Basic Veterinary Medicine, Panhe Campus, Shandong Agricultural University, Taian 271018, China; yuwenjing0224@163.com (W.Y.); zzx11012025@163.com (Z.Z.); zhwang@sdau.edu.cn (Z.W.); linxueyan@sdau.edu.cn (X.L.)

**Keywords:** mastitis, cow, diagnosis, treatment

## Abstract

Mastitis, an inflammatory disease caused by the invasion of various pathogenic microorganisms into mammary gland tissue, is a core health issue plaguing the global dairy industry. The consequences of this disease are manifold. In addition to directly compromising the health and welfare of dairy cows, it also precipitates a substantial decline in lactation function, a precipitous drop in raw milk production, and alterations in milk composition (e.g., increased somatic cell counts and imbalanced ratios of milk protein to fat). These changes result in a marked degradation of milk quality and safety, and in turn, engender significant economic losses for the livestock industry. Therefore, the establishment and implementation of a comprehensive prevention and control system is a key strategy to effectively curb the occurrence of mastitis, reduce its incidence rate, and minimise economic losses. This review systematically explores the complex etiological factors and pathogenic mechanisms of mastitis in dairy cows, and summarises various diagnostic methods, including milk apparent indicators monitoring, pathogen detection, physiological parameter monitoring, omics technologies, and emerging technologies. Furthermore, it undertakes an analysis of treatment protocols for mastitis in dairy cows, with a particular emphasis on the significance of rational antibiotic use and alternative therapies. Moreover, it delineates preventive measures encompassing both environmental and hygiene management, and dairy cow health management. The objective of this paper is to provide a comprehensive and scientific theoretical basis and practical guidance for dairy farming practices. This will help to improve the health of dairy cows, ensure a stable supply of high-quality dairy products, and promote the sustainable and healthy development of the dairy farming industry.

## Simple Summary

Mastitis in dairy cows is an inflammation caused by infection of mammary tissue by pathogenic microorganisms, which seriously affects the health of dairy cows, leading to a decline in milk production and deterioration of milk quality, and is one of the most important problems restricting the animal husbandry economy. Therefore, the establishment of a comprehensive system of ‘prevention-oriented and precise treatment’ is the core strategy to reduce the incidence of mastitis. This paper reviews the causes of mastitis, diagnostic techniques, therapeutic methods, and preventive systems to provide guidance for promoting the development of dairy farming and safeguarding the health and safety of dairy cows.

## 1. Introduction

Mastitis, a prevalent and economically detrimental disease affecting dairy cows on a global scale, is a major concern for the dairy industry. It is classified into two distinct categories based on its etiology: infectious and environmental [1]. The former is primarily attributable to infectious pathogens such as *Staphylococcus aureus* (*Staph. aureus*)*, Streptococcus agalactiae* (*Strep. agalactiae*), and *Mycoplasma*, which are disseminated among dairy cows via hands, towels, milking cups, and other implements employed during the milking process [2]. The latter is primarily attributable to environmental pathogens such as *coagulase-negative Staphylococcus* (CNS), *Escherichia coli* (*E. coli*), and environmental *Streptococcus*, which infect healthy dairy cows through feces, soil, and damp bedding [3]. Mastitis can be categorised into two distinct classifications, namely clinical mastitis (CM) and subclinical mastitis (SCM), based on the manifestation of symptoms [1]. The former is characterised by cows exhibiting fever, loss of appetite, and lethargy, with swollen, hot, and red udders, watery milk or the presence of flakes and clots, and a significantly elevated somatic cell count (SCC) exceeding 70,000 cells/mL [4]. The latter type does not exhibit overt physical, mammary, or milk characteristics in cows and can only be detected through testing; however, it is associated with reduced milk production and increased SCC [5,6].

Mastitis poses a grave threat to the welfare of dairy cows, public health, and the economic viability of the dairy industry. Cows suffering from CM experience significant discomfort and impaired reproductive performance, as evidenced by reduced conception rates (requiring more artificial insemination attempts), prolonged intervals between calving and first artificial insemination, increased abortion rates, and extended open periods. Furthermore, lactation performance is significantly diminished, as evidenced by reduced milk production and diminished milk fat content [7]. From a public health perspective, mastitis poses dual risks. Firstly, milk and dairy products from infected cows may carry zoonotic pathogens, thus threatening human health [8,9]. Secondly, the inappropriate use of antibiotics—a key control measure—can promote the emergence of antibiotic-resistant strains, thus posing a global health hazard [10,11]. From an economic perspective, the financial impact of mastitis is substantial, encompassing both direct and indirect costs. Direct costs are primarily attributable to drug treatments for clinical cases, discarded milk, and culled cattle, accounting for approximately 30% of total losses [12]. The preponderance of indirect costs is attributable to diminished milk output consequent to subclinical infections, constituting approximately 70% of aggregate losses [13]. Research indicates that the financial implications of CM cases in the initial 30 days of lactation at a designated dairy farm in the United States are estimated to be approximately $444 [14]. In India, annual losses attributable to mastitis are reported to amount to approximately $98.228 billion (716.551 billion Indian rupees) [15]. This emphasises the severe consequences of mastitis and underscores the necessity for timely diagnosis and the implementation of effective prevention and control measures.

The optimal approach for the diagnosis of mastitis should be characterised by its expeditiousness, precision, and timeliness, thereby facilitating the timely identification and management of the condition. Conventional early detection methodologies, including the California Mastitis Test (CMT) and electrical conductivity (EC) tests have been extensively adopted in agricultural practices due to their expeditious results, economic viability, and suitability for on-site implementation. Nevertheless, these methodologies exhibit comparatively low specificity. Conversely, advanced diagnostic tools such as polymerase chain reaction (PCR), enzyme-linked immunosorbent assay (ELISA), and omics technology are dependent on complex laboratory facilities and are costly. However, these tools offer the advantages of high accuracy and specificity [16,17]. A systematic review is therefore required of the advantages and disadvantages, applicable scenarios, and development trends of existing and emerging diagnostic methods. This will assist cattle farms in selecting the optimal or combined diagnostic strategies based on actual conditions and promote the development of more ideal diagnostic tools.

In the management of mastitis, a range of traditional and emerging approaches are employed for treatment and prevention, including antibiotics, vaccination, herbal therapy, bacteriocins and nanoparticle-based therapy [18]. Moreover, research has demonstrated that the utilisation of chemical disinfectants is effective in mitigating udder infections in bovines, particularly mastitis, and enhancing the quality of milk [19]. At present, antibiotic treatment remains the mainstream approach; however, its widespread use has led to the problem of antibiotic resistance, which is increasingly undermining its effectiveness [20,21]. Vaccination, as a significant preventive strategy, encounters challenges in its practical implementation. On the one hand, due to the wide variety of pathogens that cause mastitis (e.g., *Staph. aureus*, *Strep. agalactiae*, *E. coli*), although vaccine development targets have been identified, the overall success rate is low [22,23,24]. On the other hand, existing vaccines have been observed to demonstrate limited preventive efficacy and are costly [25]. In light of the limitations inherent to conventional antibiotic and vaccine treatments, alternative therapeutic modalities, including bacteriocins and nanoparticle therapy, are garnering attention due to their distinctive therapeutic potential [26,27]. Consequently, a thorough review of contemporary treatment and prevention methodologies, both those that are currently employed and those that are still under investigation, will facilitate the identification of existing approaches’ limitations, reveal the synergistic potential of diverse strategies, and delineate research priorities and optimisation directions for reducing antibiotic dependence and enhancing prevention outcomes.

Mastitis in dairy cows poses a serious risk to animal welfare, economic efficiency, and public health. Yet existing diagnostic techniques and treatments have many shortcomings. This article provides a comprehensive review of the various aspects of mastitis in dairy cows, with a particular focus on its etiology, diagnostic techniques, treatment methods, and preventive measures. A critical analysis of the advantages and disadvantages of various diagnostic techniques is presented, with a focus on the necessity of identifying alternatives to antibiotics and the potential of emerging technologies. The objective is to ensure the health of dairy cows and to mitigate economic losses.

## 2. Methods

In this study, a systematic search was conducted across three prominent academic databases (Google Scholar, Web of Science, and PubMed), with the objective of extracting literature pertaining to the themes of “cow/bovine/cattle” and “mastitis”. These core subject terms were employed in conjunction with the Boolean operators OR/AND, with the search focused on literature published within the last decade. The final inclusion criteria entailed academic results that were directly related to the subject of the review.

## 3. The Causes of Mastitis

Mastitis is a multifactorial disease, and susceptibility to it is influenced by three main factors: pathogenic microbial infection, environmental factors, and the cow’s own condition. Among these, pathogenic microbial infection is the most direct and fundamental cause of mastitis, triggering the body’s immune response and leading to infection and inflammation [28].

### 3.1. Pathogenic Microbial Infections

The most prevalent bacteria that cause mastitis include *Staph. aureus*, *Strep. agalactiae*, *Strep. pyogenes*, *Trueperella pyogenes* (*T. pyogenes*), *E. coli*, *Klebsiella pneumoniae* (*K. pneumoniae*), *Klebsiella oxytoca* (*K. oxytoca*), *Enterobacter aerogenes*, and *Pasteurella* spp. [29,30]. In accordance with the principles of epidemiology, the classification of mastitis is based on two distinct categories: infectious mastitis and environmental mastitis [31]. Among these, *Staph. aureus*, CNS, *E. coli*, *Strep. agalactiae*, and *Strep. uberis* are the most significant pathogens causing mastitis worldwide [32].

#### 3.1.1. *Staph. aureus*

*Staph. aureus* is a major pathogen that is both prevalent and harmful, and its eradication is challenging [33,34]. The bacterium commonly causes chronic, subclinical infections, significantly elevates SCCs, and results in sustained milk production losses. It primarily spreads among dairy cows through the milking process, necessitating comprehensive prevention and control measures [35]. Research indicated that *Staph. aureus* infections occurring during the first or second lactation period have a more severe negative impact on milk production than those occurring during the third lactation period or later [36]. Furthermore, bovine isolates of *Staph. aureus* has been identified as a significant causative agent of foodborne illnesses, with bulk tank milk and raw dairy products being recognised as significant vectors for its transmission to humans [37].

#### 3.1.2. *Strep. agalactiae*

*Strep. agalactiae* is unable to reproduce or grow outside the udder, but it has been demonstrated to survive for a brief period on the hands of milking personnel, milking machines and surfaces. This period of survival is sufficient for the transmission of the organism to healthy cows during the process of milking. Notwithstanding the meticulous management of herds, the introduction of infected new bovines without the implementation of appropriate isolation procedures constitutes a potential risk [38]. Nevertheless, rigorous control measures have been proven to effectively manage the disease, with its prevalence having significantly decreased in specific regions of Europe and North America [39]. This pathogen demonstrates a favourable response to antibiotic treatment, with the capacity to be eradicated from herds through the implementation of effective mastitis control measures. Such measures include the dry period therapy and teat dipping after milking [34].

#### 3.1.3. *Strep. dysgalactiae*

The nature of *Strep. dysgalactiae* as either a contagious or environmental pathogen has been a subject of debate. This pathogen has been found in almost any environment (e.g., bedding, barns) and within hosts (e.g., mammary glands, rumen, feces) [40].The condition is characterised by the onset of acute inflammation. Research has indicated that the probability of infection is substantially impacted by the standard of barn hygiene management and the configuration of milking systems. Furthermore, an increase in parity has been shown to result in a significant elevation in the risk of infection. In addition, an infection has been found to be positively correlated with high-yield genetic performance, yet negatively correlated with the SCC genetic index [41].

#### 3.1.4. *E. coli*

*E. coli* is among the most prevalent pathogens responsible for environmental mastitis. Typically, mild infections manifest solely with localised breast symptoms and alterations in milk quality, resulting in minimal breast tissue damage following recovery. Conversely, acute infections frequently culminate in severe CM, accompanied by substantial systemic symptoms (e.g., fever, loss of appetite, depression), and may even prove fatal, leading to a substantial and protracted decline in milk production [42,43].

#### 3.1.5. CNS

CNS species characteristically exhibit reduced virulence and predominantly induce mild clinical or SCM, resulting in diminished milk quality, breast tissue damage, diminished milk production, and mildly elevated SCCs [44]. Research has indicated that the prevalence of CNS is elevated in South China relative to North China, with a greater proportion of clinical mastitis cases in comparison to subclinical cases [45]. Furthermore, a study by Elhaig et al. confirmed that CNS carries the ica gene, which enhances its adherence in breast tissue, thereby increasing the difficulty of disease prevention and control, and exhibiting increasing antibiotic resistance [46].

#### 3.1.6. *K. pneumoniae*

*K. pneumoniae* is predominantly found in bedding, particularly sawdust and peat, which serve as its primary hosts. Furthermore, water and soil have been identified as potential media for its survival. The pathogen has been responsible for a decline in milk production, resulting in significant economic losses for the dairy industry [47]. Research has demonstrated that bovines afflicted with CM, a condition precipitated by *K. pneumoniae*, encounter a mean daily milk yield reduction of 4.9 kg during the week of onset, with losses persisting for multiple weeks [36]. Research by Bannerman et al. shows that cows artificially injected with *K. pneumoniae* can experience a drop in milk production of approximately 60% on the first day after infection, which may fall to around 15% of the control group on the second day, and production fails to recover by the end of the study [48]. Table 1 presents an overview of the predominant pathogens associated with bovine mastitis, as identified in recent research studies.

### 3.2. Environment and Management

The presence of pathogens is the fundamental cause of the occurrence of mastitis in bovines employed in dairy farming. Nevertheless, inadequate farm management has been demonstrated to play a pivotal role in the dissemination of pathogens within the herd, consequently leading to an escalation in the prevalence of mastitis. Environmental management constitutes a pivotal element in this context. Overheated and humid barn environments, unchanging wet and dirty bedding, and inadequate ventilation not only provide a conducive environment for the proliferation of pathogens but also compromise udder hygiene, thereby indirectly augmenting the risk of infection [125,126,127]. Conversely, ensuring the barn environment remains comfortable and hygienic has been demonstrated to be an effective measure in reducing the incidence and severity of mastitis [128].

In addition to environmental factors, improper operation during the milking process is also a significant causal factor. This encompasses aberrant functionality of milking apparatus (e.g., unstable vacuum, inappropriate pulsation) [129,130], suboptimal milking methodologies (e.g., vibration during milking, inadequate sterilisation) [131,132], and irrational milking sequence (e.g., Backward-milking cows may facing higher risk of mastitis infection) [133], all of which can directly augment the risk of mastitis.

The physiological state of dairy cows, especially during the critical stages of the transition period, has been shown to have a higher demand for nutrition and energy. In instances where the feed intake is inadequate to meet the lactation demand, the cow is susceptible to entering a negative energy balance [134]. The occurrence of a negative energy balance, associated with deficiencies in amino acids, trace elements and vitamins, has been demonstrated to result in weakening of the cow’s immune function, thereby rendering it more susceptible to mastitis [135]. Consequently, scientific management of the diet is imperative to ensure adequate energy supply and appropriate supplementation of vitamins and trace elements [136,137]. This approach is pivotal in enhancing resistance and preventing mastitis. Furthermore, other stressors, including heat stress resulting from ineffective cooling systems, within the herd, and traumatic stress arising from rough handling, have been demonstrated to compromise cow health and further increase susceptibility to mastitis [138,139,140].

### 3.3. Species and Physiological Status of Cows

Dairy cows themselves vary significantly in their resistance to pathogen attack, which is influenced by a number of factors. Firstly, breed-related genetic factors are a significant source of difference. The incidence of mastitis exhibits significant variation between breeds, with studies demonstrating that Simmental breed exhibited a higher risk of mastitis in all farming regions, with a particularly high incidence of 52.5% observed in the Mediterranean region. In contrast, the Holstein breed demonstrated the lowest prevalence, recorded at 42.8% in the Eastern region [141]. Furthermore, the study by Yunusa et al. indicated that the Bunaji breed exhibited a higher prevalence compared to the Wadara breed [142]. In addition to this, a genome-wide association study (GWAS) meta-analysis across breeds (covering six dairy breeds) further confirmed the existence of genetic heterogeneity and identified 58 genetic markers associated with mastitis resistance [143].

Secondly, the physiological stage and individual characteristics of the cow are important influencing factors. It is well established that bovines in the transition period are at significantly increased risk of developing mastitides [144]. Furthermore, age is an important factor to consider. Research has demonstrated that bovines over the age of six are more prone to the condition, a phenomenon that may be attributed to teat laxity and elevated mammary epithelial permeability resulting from protracted milking [142]. An increased litter size has been demonstrated to be associated with a higher prevalence of mastitis, with the risk increasing in accordance with lactation length, as evidenced by the study conducted by Nurye and Mramba [145,146].

Finally, the physical structure and morphology of the breast itself directly affect susceptibility to infection. Specifically, the presence of a triangular barrel and pointed teat end (TP) or a square barrel and flat teat end (SF) has been demonstrated to carry a heightened risk, while the configuration of a square barrel and round teat (SR) has been shown to be the least risky [147]. Furthermore, a high degree of breast ptosis and loose attachment were found to be associated with higher SCCs, which in turn increased the prevalence of mastitis [148,149].

### 3.4. Molecular Mechanisms of Mastitis

The pathogens responsible for mastitis are diverse and complex, and their molecular mechanisms have not yet been fully elucidated. However, research in this field has made some progress. To illustrate this, consider *Staph. aureus*, which initiates an infection through the binding of adhesins on its surface (e.g., fibronectin-binding proteins, collagen-binding proteins, clumping factor, and teichoic acid) to the extracellular matrix proteins on the surface of host mammary epithelial cells, thereby achieving adhesion [150]. Following successful adhesion, *Staph. aureus* secretes a variety of toxins (e.g., hemolysins α, β, γ, δ, and phenol-soluble regulatory peptides) [151]. This process enables the bacteria to compromise the integrity of the host cell membrane and gain access to the cytoplasm, facilitating their reproductive.

**Figure 1:**
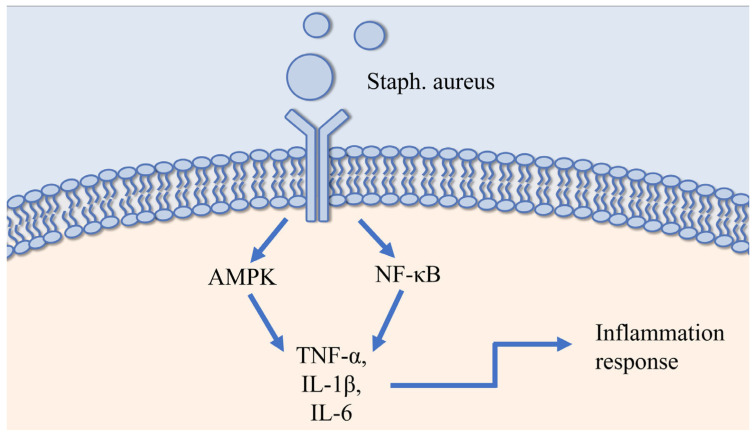
Mechanism of mastitis in dairy cows caused by *Stap. aureus*.

In the context of an immune response to infection or injury, a variety of immune cells, including neutrophils, monocytes, and macrophages, among others, play a pivotal role in the innate immune defense system. These cells are equipped with pathogen recognition receptors (PRRs) on their cell membranes, which are capable of recognizing pathogen-associated molecular patterns (PAMPs) present on invading pathogens [152]. *Staph. aureus* PAMPs primarily activate Toll-like receptors TLR2 and TLR4. Activation of TLR2/4 has been demonstrated to trigger downstream NF-κB and MAPKs signalling pathways, leading to massive expression of pro-inflammatory cytokines [e.g., tumor necrosis factor-α (TNF-α), interleukin-1β (IL-1β), IL-6] by host cells (Figure 1). These cytokines recruit and activate immune cells, such as neutrophils, to clear pathogens [153]. However, the enzymes (e.g., proteases) and reactive oxygen species (ROS) released by immune cells during the process of pathogen elimination, while acting as antimicrobial agents, can also damage mammary tissue, especially the alveolar epithelial cells. Damage of this nature has been shown to result in alterations to the composition of milk, including an increase in the SCC and a decrease in the milk fat and protein content. These changes can also lead to the development of tissue fibrosis and, in severe cases, the destruction of the alveolar structure [154].

In addition, *Staph. aureus* has evolved a variety of immune evasion mechanisms. For instance, secreted protein A has been shown to bind to the Fc segment of antibodies (IgG), thereby interfering with antibody-mediated conditioning and phagocytosis [155]. These immune evasion strategies enable *Staph. aureus* to survive and persist within the host, resulting in an infection that is difficult to eradicate and often presenting as chronic, recurrent, or SCM.

## 4. Diagnostic Methods for Bovine Mastitis

**Figure 2:**
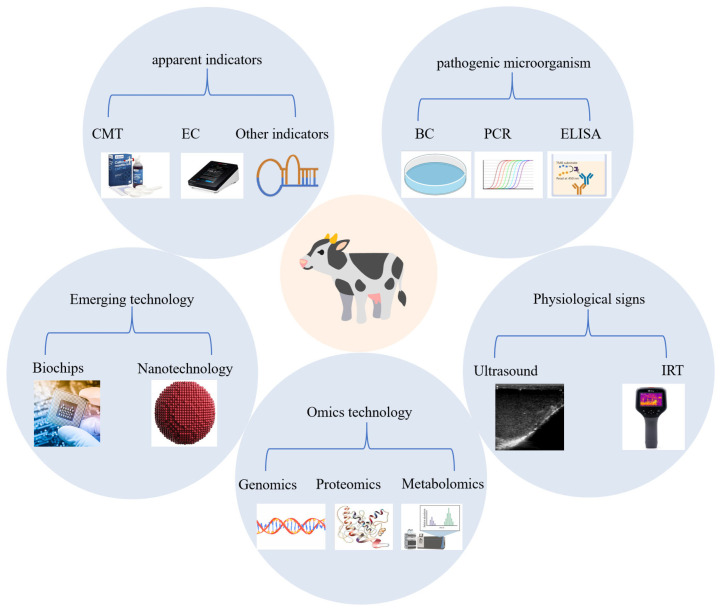
Diagnostic methods for mastitis in dairy cows.

Mastitis has been shown to have a significant impact on the welfare of dairy cows and the financial profitability of dairy farms [156]. Therefore, it is vital that the condition is diagnosed in a timely manner in order to minimise losses. The diagnosis of CM is typically made through a combination of visual inspection and palpation, with the presence of udder redness and swelling, milk abnormalities, and pain during milking being key indicators. Conversely, SCM is characterised by the absence of overt clinical manifestations and the necessity of employing specific diagnostic tools [157]. These encompass the use of SCC and other biomarker-based tests, the identification of pathogenic microorganisms, the monitoring of physiologic signs, omics technology, and the utilisation of emerging technologies (Figure 2).

### 4.1. Monitoring of Apparent Indicators in Milk

Somatic cells are cells produced by the immune system of dairy cows and migrate to mammary tissue or milk to combat infection. SCC is a significant indicator for predicting intramammary infection (IMI) and evaluating raw milk quality. Elevated SCC levels in milk have been demonstrated to be indicative of a heightened risk of IMI, in addition to exerting a direct influence on the quality of raw milk [15]. Despite the extensive utilisation of SCC for the monitoring of milk quality and the screening for IMI, there exists considerable discord regarding its diagnostic threshold. Schukken et al. proposed that an SCC ≤ 70,000 cells/mL signifies an uninfected mammary gland, whereas an SCC > 100,000 cells/mL is associated with a decline in milk production [158]. Furthermore, in a study by Karzis et al., it was stated that the optimal thresholds for SCC in composite and quartered milk samples were 150,000 and 200,000 cells/mL, respectively [159]. Despite the long-standing utilisation of SCC for the diagnosis of mastitis, it is imperative to acknowledge its inherent limitations. SCC levels are susceptible to a variety of non-infectious factors, including cow age, season, stress, feeding management, stage of labour and lactation [158]. Consequently, in recent years, researchers have dedicated themselves to the identification of other test indicators, including lactose, calcitonin, microRNA (miRNA), etc.

#### 4.1.1. CMT

CMT has been shown to destroy somatic cells in breast milk by surfactants (e.g., sodium dodecyl sulfate), resulting in the release of DNA from the cell nucleus. This process leads to the formation of a gel upon contact with the reagent. The consistency and stability of the gel are directly proportional to the number of somatic cells in the milk, thus serving as an indirect indicator of the degree of infection and inflammation in the mammary gland [160]. This method is characterised by its ease of execution, inexpensiveness and rapidity, and it is regarded as one of the most widely utilised indirect SCC methods. However, CMT is subject to certain limitations. Firstly, the results is susceptible to interference from non-infectious factors, including season, stress, and the stage of labour [158]. Although CMT is considered suitable for preliminary screening for mastitis, it is strongly recommended that it be used in conjunction with other confirmatory tests for the purpose of arriving at a definitive diagnosis. It is evident that CMT results alone are not sufficient to support major management decisions that may affect the viability and profitability of dairy farms [161].

#### 4.1.2. EC

Milk EC test is a measure of its ability to conduct electricity. In bovines afflicted with mastitis, the permeability of the blood-milk barrier (BMB) is increased, resulting in the leakage of ions such as Na^+^ and Cl^−^ from the blood into the milk, thus increasing the ionic concentration of the milk. Conductivity is proportional to the total concentration and type of ions in solution; therefore, milk EC is usually elevated in mastitis-affected cows [162]. Nevertheless, the value of using EC as a diagnostic indicator of mastitis has been the subject of considerable controversy. EC changes demonstrate no significant correlation with SCC [163]. Furthermore, EC is susceptible to interference from a variety of factors, including cow breed, season, and the stage of the lactation [164]. Consequently, the accuracy of EC in detecting mastitis is limited, and its diagnostic specificity is unsatisfactory.

#### 4.1.3. Other Test Indicators in Milk

SCC in milk has been shown to increase dramatically at the onset of mastitis, thus serving as a central marker of the condition [165]. Other biomarkers for mastitis have become a hot topic of research in recent years, with key findings from the past five years shown in Table 2. Cathelicidin (CATH), which is inflammatory peptides belonging to the innate immune system, have been found to have elevated levels prior to the onset of mastitis. Furthermore, CATH have been observed to be positively correlated with SCC [166]. Research has demonstrated that endogenous CATH is imperative for the regulation of *Staph. aureus* mammary gland infections, as substantiated by a knockout mouse model [167]. Defensins, innate immune peptides with antimicrobial and immunomodulatory effects, are up-regulated in mastitis and are important for immune defence. Furthermore, significant differences in β-defensin-4 (DEFB-4) levels were observed between local and systemic manifestations in acute CM and SCM [168].

Another significant indicator for the assessment of mastitis is the presence of acute phase proteins. Haptoglobin (Hp) is chiefly produced by the liver and its serum concentration increases as a result of bacterial inflammation and infection. It can be used to differentiate between SCM, CM, and healthy cows [169]. C-reactive protein (CRP) is classified as a major acute-phase protein, which is produced by the liver. Serum concentrations of CRP exhibit significant elevation during periods of systemic inflammation. Although the changes in milk CRP are not as pronounced in bovine mastitis as the other markers, it remains a crucial component in evaluating the extent of systemic inflammation [170]. Conversely, milk serum amyloid A (M-SAA), which is predominantly produced locally by mammary epithelial cells in response to inflammatory stimuli, exhibits a rapid and significant increase in milk concentrations during episodes of mastitis (particularly bacterial infections). It serves as a significant marker for the diagnosis of SCM and CM [171].

In relation to the composition of milk, lactoferrin (LF) has been identified as a significant innate defence factor due to its iron-binding glycoprotein properties, which exhibit potent antimicrobial and immunomodulatory activity. Concentrations of LF in milk have been shown to exhibit a substantial increase during the early stage of mastitis infection and at the SCM stage [172]. In contrast, α-lactalbumin (α-LA), a pivotal whey protein in the synthesis of lactose, functions as a marker of normal mammary gland function. Its milk concentration is typically diminished in mastitis, reflecting tissue damage and impaired lactation. This assists in differentiating between healthy and inflammatory state milk [173]. The concentration of lactose, a major component of milk, is known to decrease due to the reduced activity of mammary secretory cells as a result of inflammation. Concurrently, the presence of lactose in the blood and urine of animals with mastitis has been detected, owing to the disruption of the BMB [174]. Furthermore, the diagnostic potential of miRNAs as novel biomarkers has been a subject of considerable interest in recent years [175].

**Table 2:** Key mastitis markers studied in the last five years.

Particular Year	Biomarker	Expression in Mastitis	Description	References
2023	Procalcitonin	Upregulation	Its concentration can be used to differentiate between CM, SCM and healthy cows.	[176]
2024	miR-146a, miR-383	Upregulation	miRNA expression profiles can serve as potential biomarkers.	[175]
2021	Lactose	Downregulation	Changes in lactose content as a diagnostic method for the prevention of SCM in dairy cattle.	[174]
2021	CATH, HP, milk amyloid A(MAA)	Upregulation	They can distinguish between healthy, SCM and CM cows.	[177]
2024	CATHs, LF, HP	Upregulation	They are good biomarker candidates for early SCM.	[178]
2025	miR-148a, miR-186	Upregulation	They have the potential to be reliable biomarkers for SCM in buffaloes.	[179]
2025	Asymmetrical dimethylarginine	Upregulation	It can be a biomarker for detecting SCM and CM.	[180]
2025	miR-223-3p	Upregulation	Their diagnostic potential may be related to the different stages of mastitis.	[181]
	miR-26a-5p	Downregulation		
2024	miR-27a-3p, miR-223	Upregulation	They are potential biomarkers for SCM, especially that caused by *Staphylococcus* spp. and *Streptococcus* spp.	[182]
2022	M-SAA, HP, CATH, LF	Upregulation	They can be used as biomarkers for mastitis.	[183]
2024	CRP, Hp, MAA, LF, CATH	Upregulation	The use of multiple markers may have the potential to differentiate mastitis pathogens.	[184]
	α-LA	Downregulation		
2024	miR-361	Upregulation	They may be biomarkers of SCM.	[185]
	miR-455, miR-1301, miR-503	Downregulation		
2023	DEFB-4	Upregulation	It identifies acute mastitis and SCM in cows.	[168]
2021	IL-6, CRP	Upregulation	Their levels can be used as biomarkers to assess the severity of idiopathic granulomatous mastitis.	[170]

### 4.2. Detection of Pathogenic Microorganism

The identification of pathogenic microorganisms is a crucial step in confirming the diagnosis of mastitis. This includes bacteriological examination, molecular testing and antigen detection.

#### 4.2.1. Bacterial Culture (BC)

BC is a significant diagnostic method for mastitis, which involves the inoculation of milk samples onto culture media and subsequent incubation under appropriate conditions for 24–78 h, with the objective of determining the specific causative agent of the disease based on colony morphology [186]. While this method can obtain live bacteria and provide guidance for drug sensitivity testing, it has limitations in differentiating between healthy and diseased cows and is time consuming and requires a laboratory environment [187]. Recent studies have demonstrated that rapid on-farm tests, such as MicroMast™ and ClearMilk Test, are capable of achieving accurate diagnoses at the farm level with high levels of sensitivity and specificity [188].

#### 4.2.2. PCR

With the advent of diagnostic techniques has rendered PCR technology the optimal method for the diagnosis of mastitis, owing to its rapid, quantitative, qualitative and extensive diagnostic capabilities. The technique is employed to determine the causative agent of the mastitis by extracting nucleic acids from the sample and amplifying pathogen-specific DNA/RNA fragments [73]. PCR is a technique that can detect microbial infections in a matter of hours, with a higher level of sensitivity than traditional BC, which can take days, and thus significantly improves the process of pathogen detection [189]. Real-time quantitative fluorescent PCR (qPCR) has been shown to have several advantages over methods such as BC and conventional PCR. Firstly, it is a faster process, which allows for greater efficiency in terms of time and resources. Secondly, it is more accurate, reducing the margin of error and improving the reliability of the results. Thirdly, it is safer for the environment and operators, as it does not require the use of ethidium bromide and subsequent agarose gel electrophoresis, which can be hazardous. Finally, qPCR results are better visualised and digitised, making data sharing more straightforward [190]. The process of data sharing is rendered straightforward. Research has demonstrated that real-time fluorescent qPCR possesses 100% sensitivity and specificity in identifying mastitis pathogens [191].

#### 4.2.3. ELISA

ELISA is an immunological diagnostic method based on the principle of an antigen–antibody reaction, which has been extensively utilised in the domain of mastitis diagnosis. In recent years, the technique has been utilised in the measurement of key mastitis biomarkers [192,193]. Such biomarkers include pro-inflammatory factors, such as tumour necrosis factor and interleukins, and acute phase proteins, including Hp [194,195,196]. In comparison with conventional bacterial culture methodologies, ELISA possesses notable advantages, including its capacity for expedited, quantitative, and highly sensitive detection of host-responsive molecules that are indicative of inflammation and disease states [197]. Recent studies have demonstrated that a modified ELISA technique based on gold nanoparticles (AuNPs) significantly enhances the detection sensitivity of conventional ELISA by optimising the antigen–antibody binding efficiency. This modified ELISA has been developed for the specific purpose of detecting bovine SCM with greater precision [198].

### 4.3. Physiological Signs Monitoring

The utilisation of imaging technology facilitates the visual monitoring of mastitis in dairy cows, thereby enabling the diagnosis of the condition. Real-time observation of mammary gland conditions can assist in the timely diagnosis of mastitis including ultrasound, Doppler ultrasound, infrared thermography (IRT).

#### 4.3.1. Ultrasound

B-mode ultrasonography utilises high-frequency sound waves to penetrate the breast tissue, thereby generating gray-scale images by detecting variations in echo intensity [199]. The principle underlying the diagnosis of mastitis is predicated on the differential acoustic characteristics exhibited by normal and diseased breast tissue. Research has demonstrated that B-mode ultrasonography measurements of udder and teat morphologic parameters, echo characteristics, and changes in the size of supramammary lymph nodes can effectively differentiate between healthy cows and cows with CM [200].

Doppler ultrasound technology is a diagnostic imaging modality that superimposes hemodynamic information on ultrasound. The quantitative evaluation of blood perfusion is achieved by detecting the intensity of erythrocyte scattering signals. Given that mastitis instigates alterations in blood flow within the affected region, the utilisation of Doppler ultrasound has been proven to be a valuable diagnostic tool, enabling the timely identification of the condition [201]. The study by Risvanli et al. marked the inaugural application of colour Doppler ultrasound in the evaluation of supramammary lymph nodes in bovines. The study’s findings underscored the association between mastitis-induced lymphocytosis and the subsequent modifications in the morphology of the supramammary lymph nodes [202].

#### 4.3.2. IRT

IRT is a technique that generates thermograms by detecting the intensity of infrared radiation on the skin surface of the mammary gland. The diagnosis is based on the significant difference in udder skin temperature between healthy cows and cows with mastitis [203]. IRT, being a non-invasive, rapid, safe and sensitive method, has been shown to be sufficient for the diagnosis of mastitis when used on its own. The highest correlation coefficients between the left fore udder zone temperature (LFUT) and the right fore udder zone temperature (RFUT) and the SCCs were observed in the study of Machado et al. (r = 0.87 and 0.88, respectively) [204]. Metzner et al. reported that *E. coli* infusion-induced acute mastitis resulted in cows with udder temperatures 2.06 °C higher than those of healthy cows [205].

### 4.4. Omics Technology

In recent years, with the continuous innovation of sequencing technology, omics technology (genomics, proteomics, metabolomics) have been widely used in animal husbandry [206]. The utilisation of this technology has been instrumental in the analysis of the potential mechanisms and diagnosis of mastitis [207].

#### 4.4.1. Genomics Approaches

Genomics technology facilitates comprehensive research into mastitis in dairy cows by acquiring genomic data through methods such as genome-wide association analysis [208]. Firstly, within the domain of pathogen research, this technology has the capacity to analyse the genotypes of pathogens, thus facilitating the development of targeted prevention and control strategies. Khasapane et al. conducted a genome-wide analysis of *Staph. aureus* isolates from cows with SCM, identifying 11 different antimicrobial resistance genes and 23 virulence-related genes. These findings provide a direct basis for prevention and control programs [209]. Furthermore, Nesaraj et al. identified genomic markers for the adaptation of the *Staph. aureus* CC1/ST1 spectrum to bovine hosts for the first time. These genomic markers are critical for understanding pathogen host adaptation [210]. Secondly, with regard to the exploration of mastitis mechanisms and the mining of biomarkers, the field of genomics has the potential to facilitate the investigation of the regulatory mechanisms of mastitis and the screening of potential markers. Wang et al. undertook an integrative analysis of small RNA sequencing and DNA methylation data from cows with natural SCM, with the objective of elucidating the role of multigenomic signatures in the context of altered immune responses and impaired mammary productivity during SCM. This analysis resulted in the identification of several candidate mastitis markers [211]. Finally, in the context of genetic resistance and drug resistance studies, this technology is imperative for comprehending the genetic characteristics of mastitis and the genes associated with drug resistance. The initial GWAS to systematically evaluate traits associated with mastitis resistance in dairy cows was conducted by Narayana et al., resulting in the identification of the associated genomic regions and candidate genes [212].

#### 4.4.2. Proteomics Approaches

Proteomics technology is increasingly being utilised as a significant instrument in the realm of mastitis research, owing to its capacity to expeditiously, precisely and reliably diagnose pathogens. Among these techniques, matrix-assisted laser desorption ionization-time of flight mass spectrometry (MALDI-TOF MS) is a seminal proteomics-based technology that utilises a soft ionisation approach to analyse biological macromolecules. This technique determines the composition and structure of the substance through the measurement of the mass-to-charge ratio (m/z) of the ionised molecules [213]. In view of the advantages demonstrated by MALDI-TOF MS, recent studies have strongly suggested that it is a rapid and reliable identification method with a high potential to replace the routine identification of mastitis pathogens isolated from raw milk [214]. Lopes et al. confirmed that this method enables rapid species-level identification of *Staph. aureus* in routine laboratories [215]. Furthermore, utilising this methodology, Guimarães et al. successfully isolated and characterised six species of enterococci from 93 CM samples (5.9%) [216], while Pereira et al. employed it to group lactation-free *Streptococcus* isolates from bovines and buffaloes with SCM, and correlated the results of the grouping with antimicrobial drug sensitivity profiles [58]. Collectively, these studies demonstrate that MALDI-TOF MS is an economical and efficient tool for the identification and analysis of pathogens in the context of epidemiological studies of mastitis.

#### 4.4.3. Metabolomics Approaches

The application of metabolomics in the analysis of milk samples has seen a marked increase in research interest in recent years, driven by the continuous development and refinement of the instruments and analytical techniques related to metabolomics [217]. The application of metabolomics in the analysis of diverse body fluids, including milk, blood, rumen fluid, and urine, from dairy cows, facilitates effective monitoring of their health status and provides insights into the pathometabolic mechanisms underlying mastitis. A number of studies have confirmed the value of this technology. For instance, Yu et al. combined 16S rDNA sequencing with untargeted metabolomics analysis to compare the differences in blood parameters, intestinal microbiome, plasma and fecal metabolomes between healthy cows and cows with SCM. The results of the study demonstrated that SM cows exhibited significant intestinal dysbiosis and altered metabolic profiles. This was evidenced by decreased abundance of intestinal commensals, potential probiotics, and compounds with anti-inflammatory and antioxidant effects, and increased abundance of potentially pro-inflammatory bacteria and lipid metabolites, accompanied by oxidative stress [218]. Zhang et al. conducted a study that confirmed the efficacy of metabolomic analysis in identifying individual dairy cows susceptible to SCM at -8 and -4 weeks pre-partum. The study further suggested that the blood metabolome could be utilised for the diagnosis of SCM. Additionally, the study identified potential blood biomarkers for SCM, including lysine, ornithine, and isoleucine [219]. Furthermore, Chenglin et al. revealed that the impact of mastitis on the milk metabolome (particularly amino acids and sugars) was considerably more pronounced than its effect on the fecal metabolome, as evidenced by metabolomic comparisons. Subsequent pathway analysis further elucidated that amino acid metabolism and energy metabolism constituted the primary metabolic pathways that were subject to alteration due to mastitis [220]. In summary, metabolomics technology provides a powerful tool for a deeper understanding of the pathogenesis of mastitis.

### 4.5. Emerging Technology

The advent of emerging technologies, such as biochips and nanoparticles, has introduced a novel dimension to the field of mastitis diagnosis. These technologies have the potential to serve as a potent and efficient instrument for the precise, expeditious, and on-site diagnosis of mastitis.

#### 4.5.1. Biochips

The advent of microfluidic and biochip-based diagnostic technologies for mastitis has been observed. Such platforms facilitate portable detection through the use of magnetic materials combined with magnetoresistive sensors, thereby enabling efficient isolation, identification and enumeration of pathogens [221]. For instance, the magnetoresistive biochip utilised by the Viveiros team underscores its clinical significance by targeting the 16S rRNA gene for the concurrent detection of five pathogens associated with bovine mastitis [222]. This technology combines the advantages of high throughput, high speed, high automation, and low sample requirement; however, its promotion is still limited by the high cost of chip preparation and the reliance on sophisticated detection equipment [223].

#### 4.5.2. Nanotechnology

The advent of nanotechnology has led to the emergence of novel approaches for the diagnosis of mastitis, with these approaches leveraging the distinctive optical, magnetic and electrical properties of nanomaterials [224]. The primary benefit of the technology lies in its capacity to selectively capture target molecules or microorganisms, while effectively differentiating interfering components in complex sample matrices [225]. Chinnappan et al. developed a novel colourimetric assay for the rapid differentiation of mastitis milk from healthy milk. This was achieved by coupling magnetic nanoparticles of a fibrinolytic substrate to form a black self-assembled monolayer on the surface of a gold sensor [226]. The technology has been demonstrated to enhance detection signal and sensitivity, whilst concomitantly simplifying the process and supporting multifunctional integration. These characteristics are indicative of its potential application in the diagnosis of mastitis [227].

## 5. Treatment Methods for Bovine Mastitis

**Figure 3:**
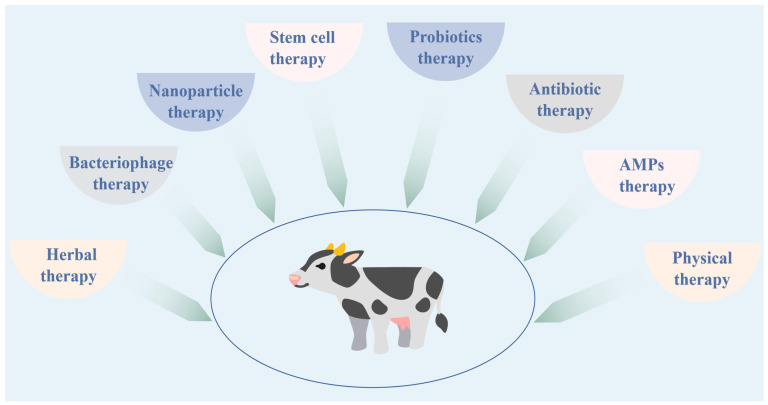
Treatment methods for mastitis in dairy cows.

It is vital important that treatment is administered promptly when cows are diagnosed with mastitis. The objective is to swiftly eradicate the pathogen, alleviate clinical symptoms, and mitigate its repercussions on the cow’s performance and well-being. The primary treatment options encompass antibiotic therapy, herbal therapy, bacteriophage therapy, antimicrobial peptides (AMPs) therapy, and nanoparticle therapy (Figure 3).

### 5.1. Antibiotic Therapy

Antibiotic therapy (including intramammary infusion, intramuscular or intravenous penicillin, ampicillin, and tetracycline) remains the primary strategy for treating mastitis in dairy cows. The selection of antibiotics should be based on a comprehensive evaluation of the patient’s history, the underlying cause of the infection, the results of drug sensitivity tests, and the established principles of therapy [228]. In recent years, research has been devoted to the optimisation of antibiotic usage in order to reduce exposure and the risk of resistance. For instance, a study by Svennesen et al. demonstrated that for mild-to-moderate CM caused by Gram-positive organisms, the bacteriologic cure rate of penicillin by intramammary injection alone was non-inferior to that of intramammary injection combined with intramuscular injection therapy. Furthermore, the daily antibiotic dosage of the former (0.6 g) was only about 1/16 of that of the latter (9.6 g), which significantly reduces the total exposure to antibiotics and the potential risk for drug resistance [229].

Nevertheless, the efficacy of antibiotic therapy still has significant limitations. Antibiotics are effective in clearing infections; however, they do not offer protection to breast tissue against irreversible damage [230]. Of particular significance is the issue of antibiotic misuse, which has given rise to a grave problem of drug resistance. Research has continued to identify and isolate multidrug-resistant (MDR) mastitis pathogens. For instance, Anika et al. have identified a MDR *E. coli* MAHK_SCM_BAU_30A strain from bovine SCM milk samples in Bangladesh [231]. Similarly, a methicillin-resistant *Staph. aureus* (MRSA) t304/ST6 strain has been identified from bovine CM. This strain was first identified by Kløve et al. [232].Consequently, the quest for safe and effective alternative antibiotic therapies has emerged as a prominent trend in dairy mastitis research.

### 5.2. Herbal Therapy

Herbs, as valuable components of traditional medicine, are attracting the interest of researchers for the treatment of bovine mastitis. In comparison with antibiotics, herbs and their extracts possess the advantage of being less prone to resistance (even with prolonged exposure) and low toxicity [233]. A plethora of herbs or their extracts have been demonstrated to possess antimicrobial properties (Table 3). Moreover, baicalein, curcumin, and resveratrol have been subject to more extensive study with regard to their mechanisms of action.

Baicalein is a flavonoid substance that is extracted from the plant Scutellaria baicalensis. In vivo studies have demonstrated that baicalein, encapsulated in chitosan, forms a triple complex with β-lactam antibiotics that significantly reduces mastitis-associated mean blood leukocyte and neutrophil counts and inhibits matrix metalloproteinase 9 (MMP-9) and CRP concentrations [234]. It has been established that baicalein combines with glucuronic acid to form a flavonoid glycoside known as baicalin. Baicalin was shown to attenuate H_2_O_2_-induced oxidative damage in bovine mammary epithelial cells (BMECs) by up-regulating downstream antioxidant genes (NQO1, HO-1) and antioxidant systems (SOD, T-AOC) through activation of the Keap1/Nrf2 signaling pathway [235]. Furthermore, a study by Mao et al. found that Lactobacillus-Cell-Free Supernatant (LAB-CFS), in combination with baicalein, reduced the levels of mastitis-associated inflammatory cytokines by controlling the activation of the NF-κB signalling pathway [236]. It is noteworthy that, despite the disparity in their origins, studies have demonstrated that astragalus polysaccharide has been observed to impede Staph. aureus colonization within the mammary gland, modulate inflammatory responses, fortify the BMB, and hinder delayed oxidative stress by calibrating the intestinal flora and short-chain fatty acid metabolism [50].

Curcumin, the primary compound of turmeric, is regarded as a potentially efficacious pharmaceutical agent for the treatment of bovine mastitis. Research has demonstrated that curcumin mitigates lipopolysaccharide (LPS)-induced oxidative stress, inflammatory response and cell death in BMECs via the NFE2L2 signalling pathway. In order to enhance the efficacy of curcumin, researchers investigated various dosage forms [237]. Nanocurcumin was found to be particularly effective in mitigating LPS-induced mastitis inflammation and oxidative stress by activating the Nrf2 pathway, while concomitantly inhibiting the TLR4-mediated NF-κB and HMGB1 signalling pathways [238]. In contrast, curcumin binds to zeolitic imidazolate framework-8 (ZIF-8) to form the complex ZIF-8@CCM, was able to attenuate Staph. aureus-induced inflammation by inhibiting the activation of TLR2-NF-κB pathway [239]. Furthermore, the combination of curcumin and quercetin exerted a positive effect on neutrophil cell migration, ROS generation, phagocytosis, bacterial killing, and neutrophil extracellular trap (NET) release [240].

Resveratrol is a naturally occurring polyphenol that is found in a variety of plant sources, including grapes, berries and peanuts. Its core mechanism of action involves the activation of the Nrf2 signaling pathway, which in turn reduces the production of pro-inflammatory cytokines and enhances antioxidant levels by counteracting the NF-κB and MAPK pathways [241]. In terms of cytoprotection, resveratrol was shown to alleviate LPS-induced apoptosis in BMECs via the PGC1α-SIRT3 axis [242]. It has been demonstrated that the activation of the PGC-1α pathway through PRKAA1 can enhance the mitochondrial antioxidant capacity, thereby reducing the inflammatory response of BMECs [243].

**Table 3:** Role of herbs and their extracts in mastitis in the last five years.

Particular Year	Herbs/Extracts	Research Target	Model Type	Finding	References
2024	Baicalin	BMECs	In vitro	It attenuates H_2_O_2_-induced oxidative damage in BMECs by activating the Keap1/Nrf2 signaling pathway and increasing the expression of the downstream antioxidant genes NQO1 and HO-1 as well as the antioxidant systems SOD and T-AOC.	[235]
2020	Matrine, Baicalin	BMECs	In vitro	Matrine decreased the protein expression levels of endogenous and exogenous cleaved caspase-3, cleaved caspase-8, and cleaved caspase-9, and baicalein down-regulated the expression of cleaved caspase-9 and inhibited apoptosis in BMECs.	[244]
2025	Baicalin	Kunming mice	In vitro and in vivo	LAB-CFS together with baicalein controls the activation of the NF-κB signaling pathway and thus reduces the levels of inflammatory cytokines associated with mastitis.	[236]
2023	Baicalin	BMECs	In vitro	It inhibits cofilin phosphorylation or Tau hyperphosphorylation by regulating the activation of RhoA/ROCK/LIMK and PI3K/AKT/GSK-3β signaling pathways.	[245]
2024	Baicalein	Swiss albino mice	In vivo	Chitosan-encapsulated baicalein in a triple complex with beta-lactam antibiotics significantly reduced mean blood leukocyte and neutrophil counts and inhibited matrix MMP-9 concentrations and CRP responses.	[234]
2025	Astragalus polysaccharide	ICR mice	In vivo	It reduces *Staph. aureus* colonization in the mammary gland by regulating intestinal flora and short-chain fatty acid metabolism, moderates inflammatory responses, protects the BMB and prevents oxidative stress retardation.	[50]
2025	Astragalus polysaccharide	Kunming mice, MAC-T	In vitro and in vivo	It reduces the EMT process through the ROS/NLRP3 signaling pathway and inhibits breast fibrosis	[246]
2022	Astragalus polysaccharide, astragaloside IV	BMECs	In vitro	They attenuate inflammation in BMECs by modulating the Wnt/β-cyclin signaling pathway.	[247]
2024	Angelica sinensis polysaccharide (ASP)	BALB/c mice	In vivo	Oral ASP may modulate the levels of intestinal metabolites by affecting the diversity and composition of the intestinal flora, thereby reducing breast inflammation and maintaining the integrity of the BMB.	[49]
2022	Lycium barbarum polysaccharides	BMECs	In vitro	It attenuates LPS-induced inflammatory response in BMECs through PPARγ/MAPK/NF-κB pathway.	[248]
2024	Ginsenoside Rg1	Holstein Friesian cows, MAC-T	In vitro and in vivo	It can attenuate BMB destruction by activating PPARγ to inhibit oxidative stress and subsequent excessive autophagy in subclinical bovine mastitis.	[249]
2024	Triterpenoid saponin	MRSA strain IID1677	In vitro	It increases the susceptibility of MRSA to β-lactam and aminoglycoside antibiotics.	[250]
2020	Curcumin	mice, murine mammary epithelial cells (MMECs)	In vitro and in vivo	It ameliorates *Staph. aureus*-induced mastitis injury by attenuating TLR2-mediated NF-κB activation.	[251]
2021	Curcumin	MAC-T	In vitro	It attenuates LPS-induced oxidative stress, inflammation and apoptosis in BMECs through the NFE2L2 signaling pathway.	[237]
2022	Curcumin	Wistar Albino rats	In vivo	Nanocurcumin alleviates inflammation and oxidative stress in LPS-induced mastitis by activating Nrf2 and inhibiting TLR4-mediated NF-κB and HMGB1 signaling pathways.	[238]
2022	Curcumin	mice	In vivo	Administration of nanocurcumin in *Staph. aureus* mastitis reduces oxidative stress markers, reverses antioxidant depletion and restores the histological structure of the mammary gland.	[252]
2025	Curcumin	BALB/c mice, BMECs	In vitro and in vivo	Curcumin binds to the ZIF-8 to form ZIF-8@CCM, which attenuates *Staph. aureus*-induced inflammation by inhibiting activation of the TLR2-NF-κB pathway.	[239]
2021	Curcumin quercetin	Dairy herds, milk sample	In vitro and in vivo	They positively affect cell migration, ROS generation, phagocytosis and bacterial killing, as well as the release of neutrophil extracellular traps (NETs).	[240]
2022	Quercetin	BMECs	In vitro	It alleviates the LPS-induced inflammatory response in BMECs by inhibiting the TLR4-mediated NF-κB signaling pathway.	[253]
2023	Quercetin	Milk sample	In vitro	The combination of it and gentamicin effectively inhibited *P. aeruginosa* and its biofilm formation and showed synergistic and additive effects.	[254]
2025	Quercetin	BALB/c mice, MAC-T	In vitro and in vivo	It attenuates *Staph. aureus*-induced inflammation in BMEC by disrupting cell adhesion and regulating CCL5 expression through the m6A-YTHDF2-dependent pathway.	[255]
2021	Forsythoside A	BALB/c mice	In vivo	It effectively inhibited LPS-induced mammary gland inflammation in mice by attenuating the activation of NF-κB and p38 MAPK signaling pathways.	[256]
2023	Forsythoside A	BMECs	In vitro	Its protective effect on BMECs after injury.	[257]
2023	Forsythoside A	MAC-T	In vitro	It regulates autophagy and apoptosis through the AMPK/mTOR/ULK1 pathway and attenuates inflammatory damage in MAC-T cells.	[258]
2023	Forsythoside A	MAC-T	In vitro	It altered the expression of spliceosome, lysosome and oxidative stress-related genes and reduced the effects of LPS on inflammation and oxidative stress in BMECs.	[259]
2024	Forsythoside A	Kunming mice, MAC-T	In vitro and in vivo	It attenuates LPS-induced inflammatory injury through PINK1/Parkin-mediated mitochondrial autophagy.	[260]
2023	Puerarin	Dairy herds, BMECs	In vitro and in vivo	It effectively reduces oxidative stress in BMECs, enhances intercellular tight junctions, and acts as an anti-inflammatory agent.	[261]
2023	Hordenine	ICR mice, EpH4-Ev	In vitro and in vivo	It alters the composition of the gut microbiota in mice, reduces the extent of inflammatory damage and upregulates the expression of tight junction proteins.	[262]
2024	*Salvia officinalis*	MAC-T	In vitro	It was able to effectively inhibit biofilm formation of *Staph. aureus* even at sub-inhibitory concentrations.	[263]
2023	Geraniol-a	Kunming mice, Holstein cows,	In vivo	It shows comparable therapeutic rates to antibiotics and significantly inhibits pathogenic bacteria and restores microbial communities while increasing the abundance of probiotics in milk.	[264]
2023	Resveratrol	Mice	In vivo	It does this mainly by combating NF-κB and MAPK mechanisms, by Nrf2 signaling activation, which reduces pro-inflammatory cytokine production and increases antioxidant levels.	[241]
2023	Resveratrol	BMECs	In vitro	It alleviated LPS-induced apoptosis in BMECs through the PGC1α-SIRT3 axis.	[242]
2025	Resveratrol	BMECs	In vitro	It activates the PGC-1α pathway via PRKAA1 and enhances mitochondrial antioxidant capacity, thereby attenuating the inflammatory response in BMECs.	[243]
2022	Tea tree oil, thymol, carvacrol	*Staph. aureus* ATCC BAA976	In vitro	The combination of the three inhibits Gram-negative bacteria and Candida albicans activity.	[265]
2022	*Quercus robur*, *Calluna vulgaris* L.	Six bovine mastitis clinical isolates	In vitro	They may be used as antimicrobial agents in bovine mastitis.	[266]
2021	*Origanum vulgare* L., *Satureja montana* L.	Holstein-Friesian cows	In vitro and in vivo	They may be the solution for mastitis treatment.	[267]
2022	Vitexin	Kunming mice, MAC-T	In vitro and in vivo	It promotes PPARγ to inhibit ROS production, increase antioxidant enzyme activity, and reduce inflammatory cytokines and apoptosis by alleviating endoplasmic reticulum stress and inactivating MAPKs and NF-κB signaling pathways.	[268]

### 5.3. Bacteriophage Therapy

As viruses that specifically infect bacteria and are harmless to humans, animals and plants, bacteriophages themselves and their derivatives (e.g., endolysins, exolysins and depolymerases) are regarded as valuable antimicrobial alternatives. It is anticipated that these measures will contribute to a reduction in the current reliance on antibiotics in agri-food production, enhance animal productivity, and safeguard the environment [269].

In a study of bovine mastitis caused by *E. coli*, Guo et al. isolated three specific bacteriophages (vB_EcoM_SYGD1/SYGD1, vB_EcoP_SYGE1/SYGE1, and vB_EcoM_SYGMH1/SYGMH1) from bovine farm effluent. A mixture of these three bacteriophages has been shown to significantly reduce the number of bacteria, somatic cells and inflammatory factors, thereby effectively relieving the symptoms of mastitis [270]. Research by Ribeiro et al. has demonstrated that the EcoM017 bacteriophage, isolated from sewage, can effectively lyse *E. coli* biofilms and reduce bacterial adhesion, offering potential for controlling *E. coli* mastitis in dairy cows [271].

The bacteriophage has demonstrated significant efficacy in the treatment of CM in dairy cows caused by staphylococci. Horiuk’s study demonstrated that the efficacy of Fagomast treatment was 71.4%, which was not significantly different from that of traditional antibiotic preparations (efficacy range 66.7–100%). Of particular significance was the finding that the milk disposal time during Fagomast treatment was on average 1.5 times shorter than that observed with antibiotic treatment [272]. In a related study, Mohammadian et al. isolated two Podoviridae phages, M8 and B4, from dairy farm effluent. These phages were capable of lysing the bacterium in specific cases, with phage M8 being able to lyse MDR bacteria in 20% of cases, MRSA bacteria in 30.8% of cases, and biofilm-producing bacteria in 10% of cases. Phage B4 was similarly able to lyse MDR and MRSA bacteria. Conversely, phage B4 demonstrated a higher efficacy in lysing biofilm-producing bacteria, with a 10% mortality rate. It is particularly noteworthy that both phages retained their lytic activity in the milk environment, resulting in a reduction of approximately 3 logs in the number of *Staph. aureus* colonies in milk after 8 h of inoculation [273]. Gill et al.’s study demonstrated that utilising a 5-day high-dose phage injection to treat subclinical *Staph. aureus* mastitis in dairy cows resulted in a cure rate of phage K monotherapy of only 16.7%, which was not significant. This finding indicates that there is an urgent need to enhance the formulation of phage K monotherapy [274].

### 5.4. AMPs Therapy

AMPs are a class of basic peptides with antimicrobial activity. The functions of AMPs are not only limited to antimicrobial activity, but also include anti-biofilm, immunomodulatory and other activities [275]. In recent years, research on AMPs has been the focus of considerable attention.

With regard to the validation of mastitis-related antimicrobial activity, Popitool et al. demonstrated that Pm11 AMPs exhibited significant in vitro antimicrobial activity against common pathogens of bovine mastitis (e.g., *E. coli*, *S. aureus*, *S. agalactiae*, and *S. uberis*) [276]. The [I^5^, R^8^] mastoparan-like peptide (MP), as designed by Orozco et al., has been demonstrated to rapidly depolarise the bacterial membrane of *Staph. aureus* and disrupt the membrane structure, ultimately resulting in cell death [277]. Furthermore, research into drug-resistant bacteria is ongoing. Shah et al. isolated multiple MDR isolates from mastitis milk samples and found that wasp venom peptide (Polybia MP-1) effectively inhibited 32% of *Staph. aureus*, 47% of *E. coli*, and up to 70% of *K. pneumoniae* MDR isolates by disrupting both the outer and inner bacterial membranes [278].

In terms of potential application in mastitis, Zhang et al. constructed a mammary tissue-specific expression vector carrying bovine-derived tracheal antimicrobial peptide (TAP) driven by primary bovine β-lactoglobulin gene (BLG) promoter, which successfully directed the expression of TAP in primary BMECs (pBMECs) and mice. Subsequent to the introduction of the vector into bovine-associated Staph. aureus-treated pBMECs and mice, respectively, significant antibacterial effects were observed in both in vitro and in vivo experiments [279]. In the study conducted by Wang et al., the efficacy of a maggot AMP was examined. The results demonstrated that the peptide effectively improved clinical symptoms, significantly reduced bacterial load and pro-inflammatory factor TNF-α levels, and attenuated histopathological damage in a mouse mastitis model induced by *Staph. aureus* [280].

In terms of screening optimisation, in a study by Cho et al., the antimicrobial activity of 16 AMPs was compared. The study found that PMAP-36, cc-CATH3, ML-CATH and PD-CATH exhibited effective antimicrobial activity against a broad spectrum of bacteria when compared with other AMPs and found that ML-CATH had the widest antimicrobial spectrum, lowest toxicity, and remained functionally stable under pH changes and high salt conditions. The study demonstrated the compound’s remarkable potential as a prospective antimicrobial agent [281]. Nevertheless, the implementation of AMPs is also subject to certain challenges. It has been demonstrated that bacteria have the capacity to develop resistance to AMPs, and there is a possibility of cross-resistance between AMPs and conventional antibiotics, which serves to limit their potential as a broad alternative to antibiotics [282].

### 5.5. Nanoparticle Therapy

Nanoparticle technology has shown significant potential as a novel antimicrobial agent and drug delivery vehicle in the field of mastitis treatment. Several types of nanoparticles have been evaluated and have shown favorable therapeutic effects [26].

Metallic nanoparticles, most notably silver nanoparticles (AgNPs), have been shown to possess potent broad-spectrum antimicrobial properties. Motrenko et al. synthesised AgNPs using coffee extracts, a method that is both environmentally friendly and effective in disrupting the cell membranes of yeasts and algae like *Prototheca* spp. and impeding their metabolic activities [283]. Lange et al. reported further confirmed that AgNPs and copper nanoparticles (CuNPs) and their mixtures were effective in destroying pathogenic bacterial biofilms, with the combination of AgNP and CuNP being the most effective [284]. AgNP have also been integrated into more complex systems to enhance functionality. For instance, Cadinoiu et al. demonstrated that chitosan/polyvinyl alcohol (CS/PVA) composite film containing silver nanoparticles and ibuprofen (IBF) exhibited significant antibacterial activity against *Staph. aureus*, whilst concomitantly promoting the healing of skin wounds. The composite membrane has been demonstrated to attenuate inflammation by decreasing cyclooxygenase 2 (COX2) expression and promote tissue repair by increasing α-smooth muscle actin (αSMA) and tumor necrosis factor-alpha-inducing protein family 8 (TNFAlP8) expression [285]. It has been demonstrated that other metal oxide nanoparticles possess excellent antimicrobial properties. For instance, Mostafa et al. synthesised titanium dioxide nanoparticles (TiO_2_ NPs) using aqueous leaf extracts of Artemisia herb Alba (A. herb Alba TiO_2_ NPs), which has therapeutic potential against MRSA, the bacterium that causes mastitis [286]. However, it is important to note that some metal nanoparticles (e.g., copper) have been observed to demonstrate toxicity issues at higher concentrations. An evaluation of the antimicrobial properties of Copper oxide nanoparticles (CuO-NPs), Triphala nanoparticles and Chitosan nanoparticles was conducted by Panchal et al., who suggested the use of triphala and chitosan nanoparticles to mitigate the toxicity of copper nanoparticles in cattle [287].

Chitosan nanoparticles (Ch-NPs) have attracted considerable attention due to their favourable biocompatibility and multifunctionality. Orellano et al. found that Ch-NPs could effectively inhibit biofilm formation of *Staphylococcus* with bactericidal effect, and the effect showed size-dependence, with the smaller the particles, the higher the efficiency [288]. In the study by Xiang et al., the encapsulation of *E. coli* outer membrane protein A (OmpA) into chitosan nanoparticles (NP-OmpA) was investigated as a vaccine, and it was demonstrated that this could result in the down-regulation of inflammation-related genes and antioxidant factors, and the attenuation of *E. coli*-induced internal organ damage [289]. In the context of enhancing the efficacy of antibiotics, Ali et al. demonstrated that polyvinylpyrrolidone-capped AgNPs (PVP-AgNP), in conjunction with eight distinct antibiotics, exhibited a substantial enhancement in efficacy against *Staph. aureus* mastitis strain with MDR profile, and oral administration is safer than the intraperitoneal (I/P) route [290]. In contrast, Yadav et al. found that ciprofloxacin-loaded chitosan nanoparticles exhibited concentration-dependent antibacterial activity, demonstrating enhanced efficacy against *Staph. aureus* in comparison to *E. coli* [291]. In the context of combating biofilms, Rivera et al. used by preparing chitosan nanoparticles (NQo) with tripolyphosphate (TPP) through the utilisation of ionic gelation, which effectively eradicated preformed biofilms of *P. aeruginosa* in a natural environment, while concomitantly impeding their subsequent formation [292]. This finding was further substantiated by Godoy et al., who demonstrated that NQo exhibited remarkable efficacy in impeding the proliferation of *Staph. aureus* and modulating the formation of its biofilm [293].

Furthermore, novel composite nanoparticles are being investigated. Selenium-tellurium-based nanoparticles (SeTeNPs) synthesised by Kosaristanova et al. have been shown to be effective in the treatment of acute bovine mastitis induced by MRSA. The particles were found to be biocompatible and did not cause adverse reactions at the site of administration [294].

### 5.6. Probiotics Therapy

Probiotics, defined as live non-pathogenic microorganisms, have been demonstrated to confer health benefits to the host. Research has demonstrated that an imbalance in gut flora can trigger bovine mastitis via endogenous pathways, thereby establishing a theoretical framework for the utilisation of probiotics to regulate the microbiota as a therapeutic modality for the disease [295]. Steinberg et al. demonstrated the efficacy of Lactobacillus in reducing the number of mastitis pathogens and demonstrated better adhesion capacity to mammary epithelial cells, suggesting that probiotics can influence the mammary microenvironment [296]. In a separate study, Izhar et al. investigated the direct infusion of *Lactobacillus plantarum* CM49 into cows with clinical and SCM. A post-treatment analysis revealed significant alterations in the composition of the milk microbiota, characterised by an increase in the abundance of the Proteobacteria, a decrease in the abundance of the Firmicutes, and a decrease in the abundance of key pathogenic genera (e.g., *Staphylococcus* spp. and *Streptococcus* spp.). These observations suggest that CM49 may enhance breast microbiota by impeding the proliferation of deleterious bacteria and fostering the proliferation of beneficial bacteria, thereby promoting breast health [297].

### 5.7. Stem Cell Therapy

Stem/progenitor cells from bovine mammary epithelium have been demonstrated to play a significant role in maintaining breast health, thus rendering them a potential therapeutic tool for the repair of structural/cytologic defects caused by mastitis [298]. Mesenchymal stem cells (MSC) are non-specialised pluripotent cells with self-renewal, multidirectional differentiation capacity and tissue regeneration potential. Consequently, MSC have recently attracted attention for their therapeutic potential [299].

A plethora of studies have demonstrated the efficacy of multiple sources of MSC in combating mastitis in dairy cows. A study conducted by Pokorska et al. revealed that allogeneic stem cells derived from bone marrow (BMSC) and adipose tissue (ADSC) exhibited a substantial reduction in total bacterial counts (including *Staph. aureus* and *Enterobacteriaceae* bacteria) and SCCs in milk [300]. The study of Ghai et al. utilised umbilical cord-derived MSC (UCB-MSC) and its extracellular vesicles (UCB-MSC-EV) for the treatment of mastitis. The results demonstrated a substantial decrease in SCC to safe levels in the stem cell-treated group in comparison to the antibiotic-treated group. Additionally, a notable up-regulation of anti-inflammatory cytokine, AMP, and angiogenic gene expression in stem cell-treated group was observed, indicating that UCB-MSC therapy may be more efficacious than antibiotic treatment in ameliorating SCM in dairy cows [301].

With regard to combination therapy, a study by Qin et al. further compared the effects of caffeic acid (CA) and umbilical cord mesenchymal stem cells (UC-MSCs). It was observed that both cell types were capable of attenuating the inflammatory response and pathological damage to breast epithelial cells and tissues. However, UC-MSCs were found to demonstrate superior efficacy in this regard. Of particular significance was the finding that the combination of CA and UC-MSCs was more effective than either treatment alone. The mechanism by which this occurs is due to the fact that both block the expression of downstream pro-inflammatory factors by inhibiting the p38-MAPK/NF-κB↔TNF-α signaling transduction loop, thereby alleviating mastitis [302].

In terms of safety, Peralta et al. demonstrated that injection of adipose tissue-derived MSC (AT-MSC) into the udder of dairy cows was effective in killing *Staph. aureus* in the udder with no observed side effects [303].

### 5.8. Physical Therapy

In recent years, there has been an increased focus on exploring alternative treatment options to antibiotics. Physical therapy has emerged as a significant area of interest in this regard, encompassing various forms of therapy such as acoustic plus therapy (APT), laser radiation therapy, and photodynamic therapy (PDT).

#### 5.8.1. APT

It is customary for APT to combine low-intensity ultrasound with other interventions (e.g., delivery of pharmaceuticals or mechanical stimulation) has been demonstrated to alleviate the symptoms of mastitis by improving local circulation, promoting dissipation of inflammation, and enhancing tissue permeability [304]. Leitner et al. demonstrated that in cases of clinical infection in cows, APT treatment resulted in a significant enhancement in recovery rates (67.8% vs. 35.6%), a substantial reduction in culling rates (6.8% vs. 32.2%), and an augmentation in milk production (+3.9 L/cow/day) in comparison with antibiotic treatment [305]. This conclusion is further substantiated by the study of Blum et al. in Israeli cows, which demonstrated that the highest cure and recovery rates (67.1% and 64.6%, respectively) were observed in the group of cows with CM. Furthermore, APT has been shown to offer significant financial benefits, with potential savings of up to $15,106/year in a 100-cow herd [306]. Merin et al. have reported further corroborated the merits of APT, highlighting enhanced recovery rates, augmented milk output, diminished antibiotic treatment expenses, and augmented revenue from curtailed involuntary culling [307]. Consequently, APT is regarded as a viable and sustainable alternative to antimicrobial therapy for mastitis and merits further research and promotion.

#### 5.8.2. Laser Radiation Therapy

This treatment employs low-power density laser irradiation to promote cell metabolism, provide anti-inflammatory and analgesic effects, accelerate tissue repair and regeneration, improve microcirculation, and modulate immune function [308]. The study of Moreria et al. demonstrated in a rat model that low-intensity laser light improves tissue recovery, promotes collagen type I expression, and modulates the inflammatory response [309]. In the context of mastitis, Favaretto et al. compared laser photobiomodulation therapy (PBM) combined with antibiotic therapy with antibiotic therapy alone in CM. The findings demonstrated that the combination group exhibited a substantial decline in SCC, accompanied by a 22% augmentation in milk output. In contrast, the antibiotic-only group experienced a 5% reduction in milk production. This observation underscores the synergistic impact of laser therapy when employed as an adjunctive treatment modality [310].

#### 5.8.3. PDT

PDT employs photosensitisers at particular wavelengths to yield cytotoxic ROS, which selectively eliminate pathogenic microorganisms and diseased cells [311]. Silva et al. evidenced the bactericidal effect of PDT on pathogenic bacteria responsible for SCM in dairy cows (e.g., negative coagulase *Staphylococcus*, *Streptococcus* spp.) [312]. Ranulfo et al. investigated the potential of nanocarrier-mediated delivery of photosensitisers, specifically chlorophyll extract (CHL) and curcumin (CUR), in conjunction with LED light. The results demonstrated that this approach led to a substantial reduction in teat microbial load and the maintenance of milk quality, thus providing a novel non-antibiotic strategy for the management of mastitis [313].

## 6. Preventive Measures for Bovine Mastitis

In response to clinical outbreaks of mastitis in dairy cows, timely treatment can alleviate the suffering of affected animals and mitigate economic losses. However, in order to achieve healthy and sustainable development and to maximize the benefits of dairy farming, it is crucial to establish a systematic defence system with a focus on prevention. This necessitates concerted efforts in the domains of environmental and hygiene management, as well as in the realm of dairy cow health management.

### 6.1. Environmental and Hygiene Management

It is evident that environmental management constitutes the basis for the prevention of mastitis in dairy cows. It is imperative to ensure that the environment in the barn is maintained in a clean, dry and comfortable condition in order to enhance udder health [314]. For instance, research by Duniere et al. confirmed that the utilisation of bacterial conditioners (Manure Pro, MP) can effectively enhance bedding quality and bacterial composition, thereby promoting cow hygiene, reducing milk SCCs, and ultimately preventing mastitis [315]. Recent studies have indicated that the alleviation of heat stress can contribute to the mitigation of mastitis [138].

The proper setup and maintenance of milking equipment are critical components in the prevention and control of mastitis. Mikhalev and Zimnikov’s research provides unequivocal evidence that inadequate vacuum pressure in automatic milking equipment can have deleterious consequences. Their findings indicate that milking time increases by 1.6–2.0 times, the number of cows requiring additional stimulation increases by 2.5–4.0 times, the incidence of SCM increases by 3.0–4.2 times, and the incidence of CM surges by 3.2–11.8 times. Concurrently, there is a corresponding increase in milk SCCs by 2.0–3.2 times. It is imperative to implement regular maintenance and calibration procedures for milking equipment to ensure vacuum fluctuations are meticulously regulated within the optimal range of 2–3 kPa. This approach is instrumental in averting the onset of mastitis [129].

The standardisation of operator procedures and hygiene management are equally indispensable. Evanowski et al. provide compelling evidence that targeted training for milking parlor personnel in the cleaning and disinfection of teat cups, in conjunction with the utilisation of dry towels treated with detergent and bleach, can lead to a substantial reduction in the prevalence of spore-forming bacteria in bulk tank raw milk. In light of these findings, it is recommended that farms place a priority on the enhancement of employee training and the strict enforcement of udder hygiene management procedures prior to the installation and utilisation of milking equipment.

### 6.2. Cow Health Management

The most effective method of preventing mastitis in dairy cows is the implementation of a systematic health management programme for dairy cows. The present study principally encompasses two aspects: firstly, focusing the factors associated with the cows themselves; and secondly, implementing the measures of proactive intervention.

#### 6.2.1. Host Factor

It is evident that cattle breed, parity order (PO), days in milk (DIM), and milking frequency (MF) are all pivotal factors influencing the risk of mastitis. As demonstrated by Curone et al., research findings suggest that, in comparison to high-yielding Holstein Friesian cows, Italian native breed Rendena (REN) cows demonstrate enhanced mastitis resistance during the transition period. This finding indicates that a balanced approach is necessary, integrating breeding and production management strategies that consider both milk yield (MY) and disease resistance traits. This approach is crucial to ensure the optimal health of cows while maintaining high yields [316]. Schunig et al. further analysed the effects of MF, PO, DIM, and MY on SCC and SCM, highlighting that cows with parity ≥ 4 should be prioritised for prevention and control, and herds with DIM > 305 days require enhanced monitoring and timely adjustments to milking frequency or nutritional strategies. The study also confirmed that milking three times daily effectively reduces SCC and SCM risks [317]. Furthermore, effective management of the dry period is imperative in preventing the onset of mastitis. Jukna et al.’s research indicated that a comprehensive dry period of 40–70 days is optimal, during which *Strep. agalactiae* and *Staph. aureus* are detected at their lowest levels. This maximises subsequent milk production and improves udder health [318].

#### 6.2.2. Intervention Measures

Vaccines have long been a traditional measure for preventing mastitis in dairy cows, and research in this area continues to attract attention and make progress. Recent studies have demonstrated the efficacy of various vaccine strategies. For instance, Vidlund et al. administered three subcutaneous injections of *Staph. aureus* surface-associated protein (SCSP) vaccine to dairy cows at 60, 40, and 20 days prior to expected calving. The findings indicated that this treatment regimen led to a substantial decrease in the prevalence of subclinical *Staph. aureus* mastitis at both the overall and udder level [319]. Ivanov et al. administered their vaccine in two doses, 55–70 days and 25–30 days prior to calving, respectively. This dual-dose regimen was found to successfully increase antibody levels against *Staph. aureus* [320]. In a separate study, Zeng’s team administered the keyhole limpet hemocyanin-enterotoxin (KLH-Ent) vaccine to dairy cows on days 0, 20, and 40 of the dry period. This vaccine successfully induced high levels of *E. coli*-specific antibodies, indicating that it has good potential for controlling *E. coli* mastitis [321].

In addition to direct vaccination, enhancing the immune system of dairy cows or improving the health of the mammary gland through the administration of specific additives has also been proven to be an effective complementary approach to preventing mastitis [135]. Urakawa et al. found that continuous feeding of *Bacillus subtilis* C-3102 from approximately one month before calving until the end of the entire lactation period effectively reduced the incidence of mastitis, SCC, and the average number of days of milk discarded due to mastitis [322]. Gao’s team concurred with this direction, observing that the administration of Lactobacillus, yeast, or a mixture of Lactobacillus and maltodextrin assisted in the optimisation of the composition of the mammary gland microbiome, thereby enhancing the mammary gland’s resistance [323]. In addition, Poindexter’s team confirmed that the administration of 25-hydroxyvitamin D3 to lactating dairy cows can increase serum mineral concentrations and regulate mammary gland immune function [324].

The efficacy of herbal medicines and their extracts in the treatment of mastitis has been well-documented, with significant benefits being demonstrated in clinical trials. In addition to their therapeutic applications, these products also possess unique value in preventative measures. The Aguiar team discovered that the antiseptic ointment based on green propolis and aloe vera, due to their excellent antibacterial and wound-healing properties, can effectively prevent mastitis [325]. The research undertaken by the Silva team concentrated on pomegranate extract (*Punica granatum* L.), the mechanism of action of which involves the disruption of bacterial cell membranes. When employed in pre- and post-dipping procedures, it can be efficacious in the prevention and control of mastitis in dairy cows [326]. An in-depth study was conducted by Chen’s team on the preventive mechanism of vitexin, the results of which indicate that it activates Peroxisome Proliferator-Activated Receptor γ (PPARγ) to inhibit the production of ROS, enhance the activity of antioxidant enzymes, and reduce the release of inflammatory factors and cell apoptosis by alleviating endoplasmic reticulum stress and inhibiting Mitogen-Activated Protein Kinases (MAPKs) and NF-κB inflammatory signalling pathways. Thus, it can be concluded that vitexin is an effective means of preventing mastitis caused by *Staph. aureus* [268].

Technological advances have facilitated the development of novel methodologies for the prevention of mastitis. The Mathew team investigated the synergistic antibacterial effects of zinc oxide nanoparticles (ZnONPs) and lemongrass essential oil (LGEO) when utilised as a teat sealant during the dry period, thereby demonstrating their efficacy in preventing mastitis [327]. The Kalińska team conducted a study on the potential of silver and copper nanoparticles as novel biocides for use in teat disinfection procedures before and after milking, which can prevent mastitis or reduce the incidence of SCM [328]. With regard to the implementation of intelligent monitoring and the provision of early warning systems, the research team at Feng have developed an IoT-based framework for the sensing of social behaviour in cattle herds. By simulating the transmission dynamics of mastitis in dairy cows, they were able to infer individual infection risks. These risks were then combined with SCC testing, thus providing new technical means for the early detection and prevention of mastitis in dairy cows [329].

## 7. Conclusions

Mastitis, a prevalent ailment within the global dairy industry, significantly impacts the health of dairy cows, resulting in diminished milk output, deteriorated milk quality, and diminished production performance. This, in turn, engenders substantial economic losses. Whilst existing diagnostic technologies offer the potential for early detection, their high costs limit their adoption on farms, highlighting the urgent need for the development of cost-effective, user-friendly diagnostic methods that combine high sensitivity and specificity. With regard to the prevention and control of pathogenic microorganism, the widespread use of antibiotics has been demonstrated to be an effective measure. However, this has also resulted in the emergence of severe bacterial resistance, which has led to a need to expedite the exploration of safe and effective alternative strategies. This paper summarises the findings of research which indicates that herbal remedies, phages, and probiotics show promising potential. In summary, research in the field of mastitis prevention and control requires further development. On the one hand, there is a need to continue the development of low-cost, high-precision, on-site rapid diagnostic technologies. On the other hand, there is a need to increase efforts to develop alternative therapies to antibiotics and verify their long-term efficacy and safety. The overarching objective for the future is to achieve precise early diagnosis at the farm level and to integrate it with targeted treatment based on the characteristics of pathogenic microorganisms. This will establish a more efficient and sustainable comprehensive prevention and control system for mastitis.

## Figures and Tables

**Table 1:** The main causes of mastitis in dairy cows in the past three years.

Pathogen Genus	Name of Pathogen	References
*Staphylococcus* spp.	*Staph. aureus*	[49,50,51,52,53]
	CNS	[45,46,54,55,56]
*Streptococcus* spp.	*Strep. agalactiae*	[57,58,59,60,61]
	*Strep. uberis*	[62,63,64,65,66,67,68,69,70,71]
	*Strep. dysgalactiae*	[72,73,74,75]
	*Strep. parauberis*	[71]
	*Aerococcus viridans*	[76,77,78]
*Enterobacteriaceae* spp.	*E. coli*	[79,80,81,82,83]
	*K. pneumoniae*	[84,85,86,87,88]
	*K. oxytoca*	[88,89,90]
	*Enterobacter cloacae*	[89,90]
	*Serratia marcescens*	[88,91,92]
*Pseudomonas* spp.	*Pseudomonas aeruginosa (P. aeruginosa)*	[93,94,95,96]
*Corynebacterium* spp.	*Corynebacterium* *bovis*	[97]
*Bacillus* spp.	*Bacillus cereus*	[98]
*Lactococcus* spp.	*Lactococcus lactis*	[99,100]
	*Lactococcus garvieae*	[101,102,103,104]
*Mycoplasma* spp.	*Mycoplasma* spp.	[105,106,107,108,109]
*Actinomyces* spp.	*T. pyogenes*	[110,111,112,113]
*Fungi* spp.	*Yeasts* spp.	[114,115,116,117,118]
	*Mold* spp.	[117,119]
*Algae* spp.	*Prototheca* spp.	[120,121,122,123,124]

## Data Availability

No new data were created or analysed in this study. Data sharing is not applicable to this article.
